# MRI-Based Diagnosis of Neurocysticercosis with Coexisting Cervical and Lumbar Disc Pathology: A Case Report

**DOI:** 10.15388/Amed.2025.32.2.20

**Published:** 2025-12-30

**Authors:** Sandeep Yadav, Vrinda, Pankaj Kumar, Santosh Kumar Yadav, Dheeraj Kumar

**Affiliations:** 1 GSVM Medical College, PMSSY Hospital, Kanpur, Uttar-Pradesh, India; 2 GSVM Medical College, PMSSY Hospital, Kanpur, Uttar-Pradesh, India; 3 School of Health Sciences, Chhatrapati Shahu Ji Maharaj University, Kanpur, Uttar-Pradesh, India; 4 School of Health Sciences, Chhatrapati Shahu Ji Maharaj University, Kanpur, Uttar-Pradesh, India; 5 School of Health Sciences, Chhatrapati Shahu Ji Maharaj University, Kanpur, Uttar-Pradesh, India

**Keywords:** neurocysticercosis, myocysticercosis, cervical disc disease, lumbar disc pathology, MRI, neurocisticerkozė, raumenų neurocisticerkozė, tarpslankstelinio disko liga, juosmeninio disko patologija, MRT

## Abstract

Neurocysticercosis (NCC), a parasitic infection of the central nervous system caused by *Taenia solium* larvae, is a leading cause of acquired epilepsy in endemic regions. This case describes a 40-year-old male who presented with chronic headache, dizziness, and a recent seizure episode. He had no prior history of systemic illness, tuberculosis, or neurological disorders. Magnetic Resonance Imaging (MRI) of the brain revealed multiple ring-enhancing lesions with an eccentric scolex in the cerebral and cerebellar hemispheres, accompanied by surrounding edema, confirming the diagnosis of neurocysticercosis. Additional lesions in extraocular, facial, and tongue muscles were consistent with myocysticercosis, an uncommon but clinically significant manifestation that may mimic inflammatory or neoplastic processes.

Spinal imaging demonstrated degenerative changes, including diffuse cervical disc bulges at multiple levels (C3–C7) and lumbar disc pathology at L4–L5, causing anterior thecal sac indentation but without cord compression or myelomalacia. While the spinal findings were incidental, they were clinically relevant as contributors to chronic pain and potential neurological deficits.

The coexistence of disseminated neurocysticercosis with muscular involvement and early degenerative spinal disease highlights the importance of comprehensive evaluation in patients presenting with seizures and persistent headache. Treatment included albendazole-based antiparasitic therapy, corticosteroids to reduce perilesional edema, antiepileptic medications, and conservative management for disc disease.

This case underscores the role of MRI in identifying pathognomonic features of cysticercosis, emphasizes the need for systemic evaluation, and demonstrates the significance of recognizing coexisting pathologies for tailored multidisciplinary management.

## Abstract (it)

Neurocisticerkozė (NCC) – centrinės nervų sistemos parazitinė infekcija, kurią sukelia *Taenia solium* lervos, yra pagrindinė įgytos epilepsijos priežastis endeminiuose regionuose. 40 metų vyras kreipėsi dėl lėtinio galvos skausmo, galvos svaigimo ir neseniai patirto priepuolio. Jis teigė neturėjęs sisteminių ligų, tuberkuliozės ar neurologinių sutrikimų. Magnetinio rezonanso tomografija (MRT) parodė smegenyse esant daugybę žiedinių pažeidimų su skoleksu smegenų ir smegenėlių pusrutuliuose, kartu buvo aplinkinių edemų, patvirtinančių neurocisticerkozės diagnozę. Papildomi pažeidimai akies, veido ir liežuvio raumenyse atitiko raumenų cisticerkozę, retą, bet klinikinę reikšmę turintį simptomą, kuris gali imituoti uždegiminius ar neoplastinius procesus.

Stuburo vaizdinimas parodė degeneracinius pokyčius, įskaitant difuzines kaklo disko išvaržas keliuose lygiuose (C3–C7) ir juosmens disko patologiją L4–L5, sukeliančią priekinio nugaros smegenų maišelio įdubimą, bet be nugaros smegenų suspaudimo ar mielomaliacijos. Nors stuburo radiniai buvo atsitiktiniai, jie klinikiškai reikšmingi kaip prisidedantys prie lėtinio skausmo ir galimų neurologinių sutrikimų.

Kai yra išplitusi neurocisticerkozė kartu su raumenų pažeidimu ir ankstyva degeneracine stuburo liga, ypač svarbu atlikti pacientų, kuriems pasireiškia traukuliai ir nuolatinis galvos skausmas, išsamų vertinimą. Gydant mūsų pacientą taikyta antiparazitinė terapija albendazolo pagrindu, skirta kortikosteroidų edemai mažinti, antiepilepsinių vaistų ir konservatyvus tarplastelinio disko ligos gydymas.

Šis atvejis rodo MRT svarbą nustatant cisticerkozės patognominius požymius, akcentuojama sisteminio vertinimo būtinybė ir kaip svarbu atpažinti kartu pasireiškiančias patologijas, kad būtų galima parinkti individualų daugiadisciplininį gydymą.

## Introduction

Neurocysticercosis (NCC) is a parasitic infection of the central nervous system caused by the larval form of *Taenia solium*. It is one of the leading causes of acquired epilepsy in developing countries and continues to be a major public health concern in India and other endemic regions [1]. The disease can affect the brain, spinal cord, and sometimes skeletal muscles, producing a wide range of neurological symptoms depending on the number and location of cysts.

*Magnetic Resonance Imaging* (MRI) remains the most sensitive imaging tool for detecting the characteristic features of NCC, including ring-enhancing cystic lesions with an eccentric scolex and perilesional edema [2]. It also assists in distinguishing NCC from other intracranial pathologies such as tuberculoma, abscess, or metastasis [3]. The present case report highlights a 40-year-old male with disseminated neurocysticercosis and coexisting cervical and lumbar disc pathology. It emphasizes the diagnostic value of MRI in identifying both parasitic and degenerative changes and underlines the importance of a multidisciplinary approach for accurate diagnosis and comprehensive management.

## Case Presentation

A 40-year-old male presented to the neurology clinic with complaints of persistent headache for approximately six months and recurrent dizziness for the past three months. He also experienced a generalized seizure episode on 18 April 2025, which prompted detailed neurological evaluation. The patient had no prior history of systemic illness, tuberculosis, head trauma, or familial neurological disorders. On examination, he was conscious and oriented, with no focal neurological deficit.

**Figure 1:**
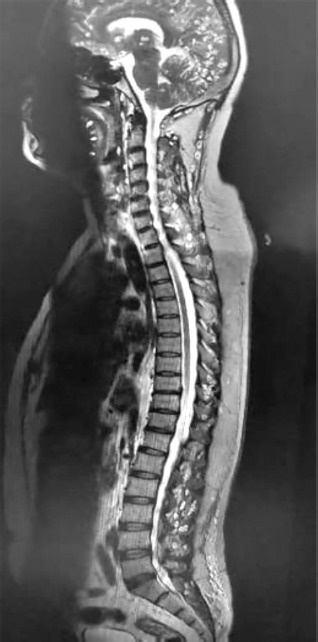
**Description:** Sagittal T2-weighted MRI of the cervicothoracic and lumbar spine demonstrates reversal of cervical lordosis with diffuse disc bulges at multiple cervical levels causing anterior thecal sac indentation. The spinal cord appears normal in caliber and signal intensity, without evidence of focal myelomalacia. The lumbar spine reveals disc desiccation with diffuse disc bulge at the L4–L5 level, producing anterior thecal sac indentation. No abnormal intramedullary signal changes or compressive myelopathy are observed. **Clinical Significance:** These findings are consistent with degenerative disc disease involving both the cervical and lumbar spine. Recognition of disc pathology at multiple levels is crucial for guiding conservative versus interventional management and for ruling out early compressive myelopathy. **Origin:** @GSVM Medical College, PMSSY Superspeciality Hospital, Kanpur, Uttar Pradesh, India. Pincode: 208002.

Magnetic Resonance Imaging (MRI) of the brain demonstrated multiple well-defined ring-enhancing lesions with eccentric mural nodules representing the scolex in the bilateral cerebral and cerebellar hemispheres, accompanied by surrounding edema – which are findings highly suggestive of neurocysticercosis (Figure 1) [4].

**Figure 2:**
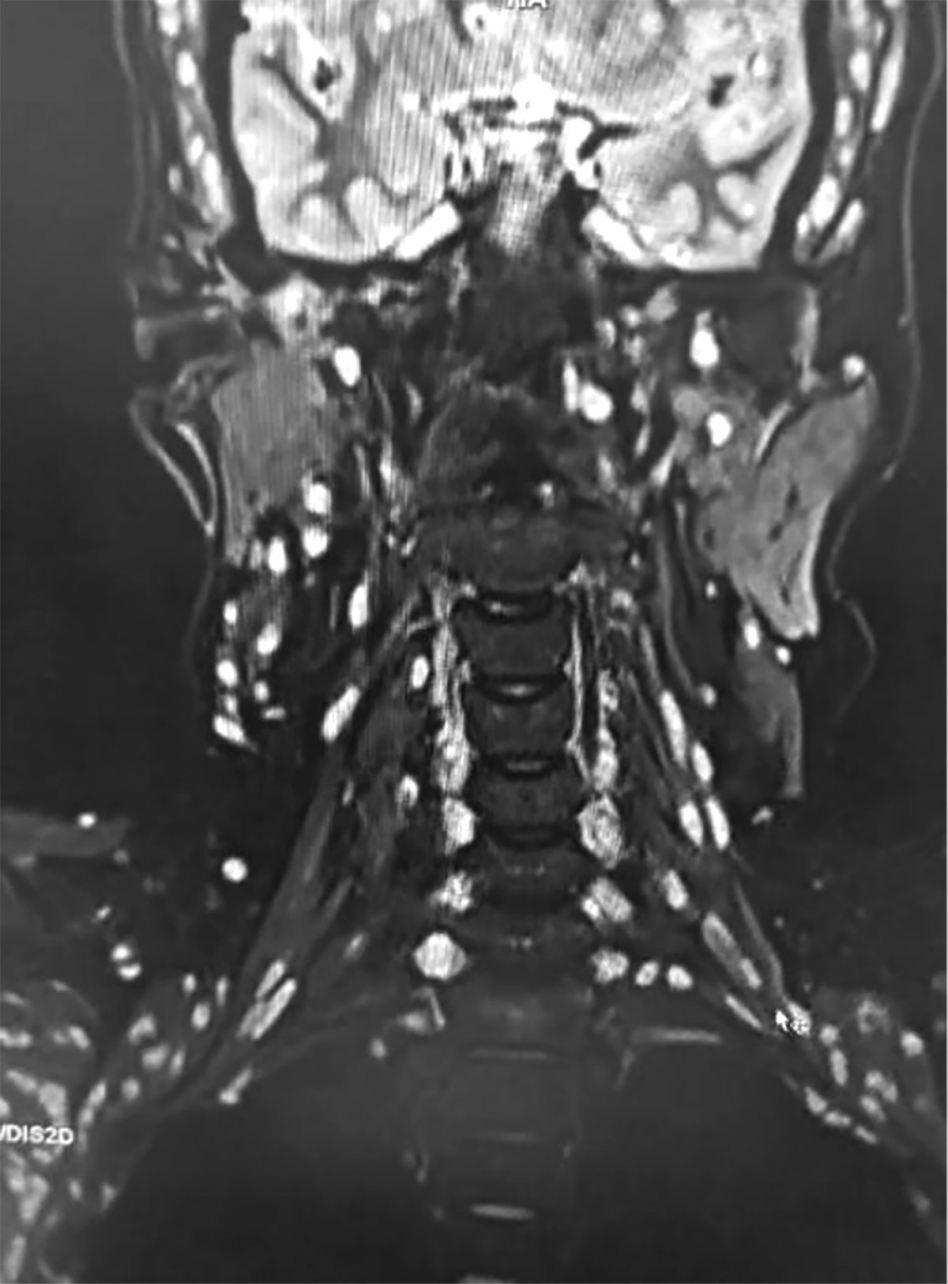
**Description:** Coronal T2-weighted MRI of the cervical spine and neck muscles shows multiple and well-defined cystic lesions distributed symmetrically within the paravertebral and posterior cervical musculature. Several lesions demonstrate an eccentric hyperintense focus representing the scolex, consistent with myocysticercosis. The cervical vertebral bodies and intervertebral discs are preserved, and no compressive myelopathy is seen. Findings are characteristic of disseminated muscular cysticercosis involving the cervical region. **Clinical Significance:** This imaging highlights the disseminated muscular form of cysticercosis, which may coexist with neurocysticercosis. Recognition of scolex within cysts on MRI is pathognomonic, allowing differentiation from other soft tissue cystic lesions or abscesses. Preservation of vertebral integrity and absence of cord compression emphasize the importance of systemic antiparasitic therapy over surgical intervention in this case. **Origin:** @GSVM Medical College, PMSSY Superspeciality Hospital, Kanpur, Uttar Pradesh, India. Pincode: 208002.

**Figure 3:**
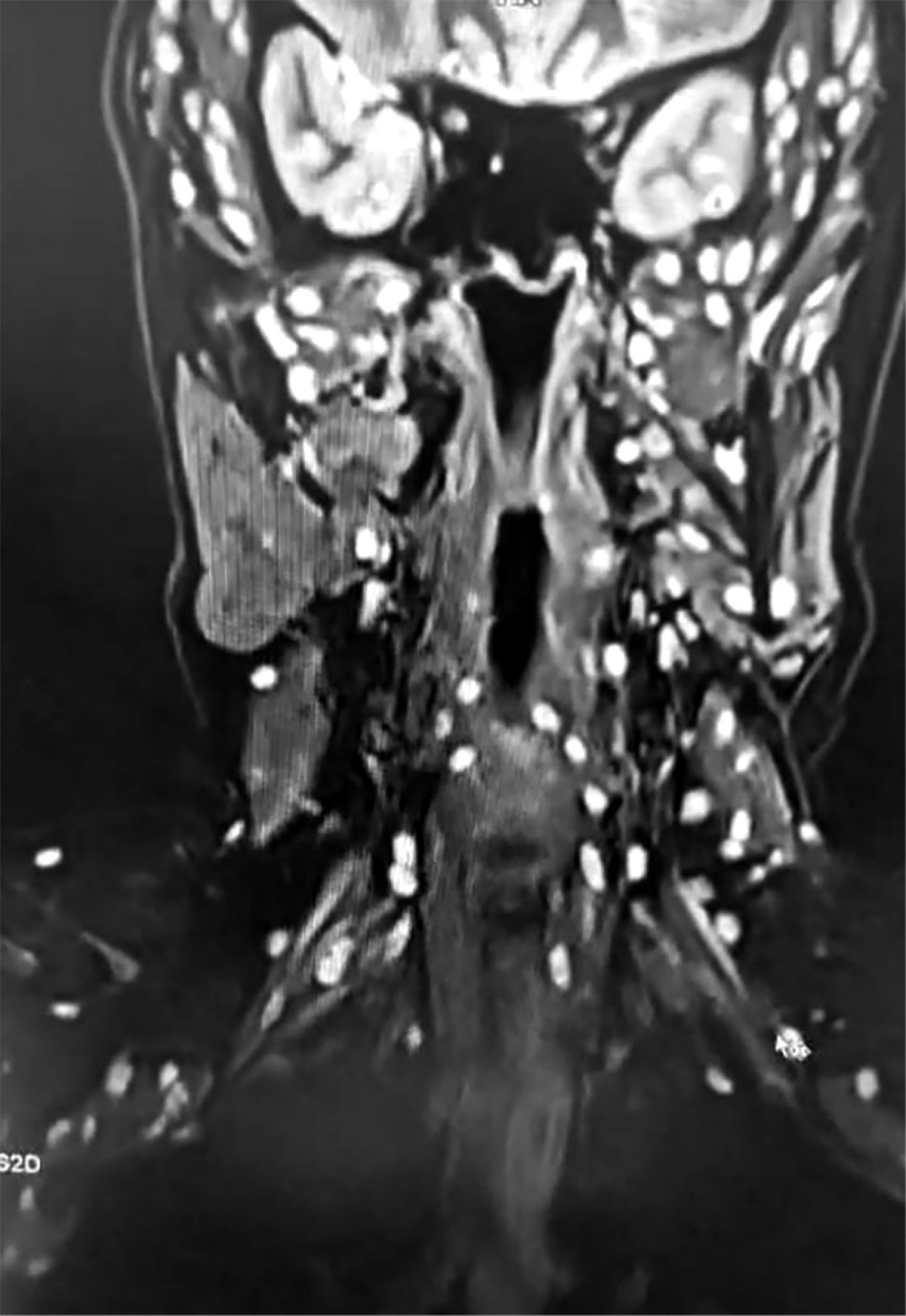
**Description:** Coronal T2-weighted MRI of the abdomen and retroperitoneum reveals numerous well-defined cystic lesions distributed along the paravertebral and retroperitoneal regions, which is consistent with disseminated cysticercosis. Multiple lesions with eccentric scolex are evident, involving muscle planes and soft tissues. Kidneys are seen symmetrically in the upper field with preserved corticomedullary differentiation. The imaging features are characteristic of systemic cysticercosis with muscular and subcutaneous involvement. **Clinical Significance:** The presence of multiple cysticerci within retroperitoneal and paravertebral regions confirms disseminated cysticercosis, an uncommon but clinically significant form of the disease. Identification of the scolex within lesions differentiates cysticercosis from other parasitic or neoplastic cystic pathologies. Preservation of renal morphology suggests no secondary renal involvement, underscoring the systemic muscular and subcutaneous distribution pattern. **Origin:** @GSVM Medical College, PMSSY Superspeciality Hospital, Kanpur, Uttar Pradesh, India. Pincode: 208002.

In addition, multiple cystic lesions were observed within the extraocular, facial, and tongue muscles, each containing an eccentric scolex, consistent with disseminated myocysticercosis (Figures 2 and 3). These lesions exhibited hyperintense signals on T2-weighted and hypointense signals on T1-weighted sequences, confirming their parasitic nature.

**Figure 4:**
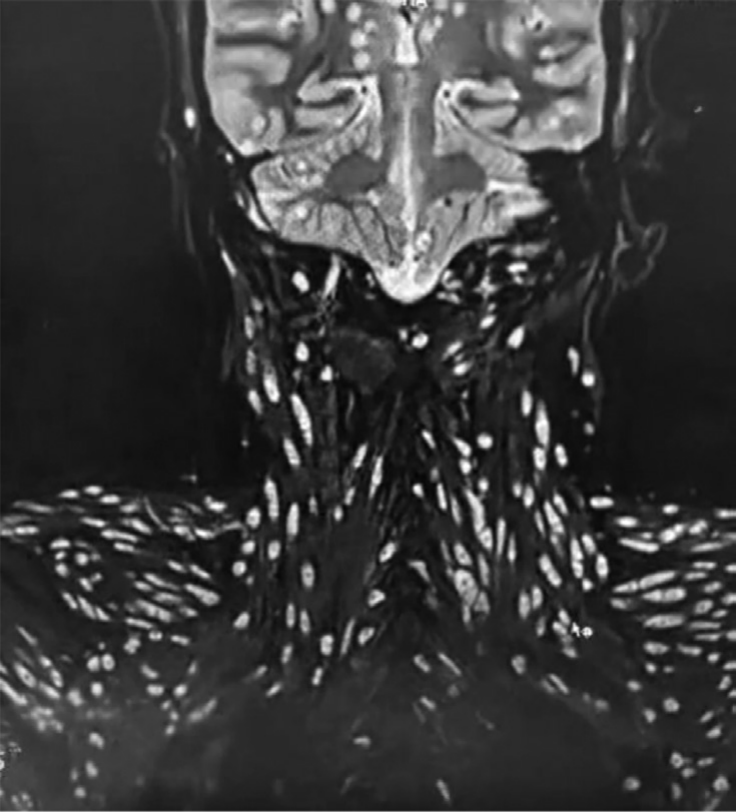
**Description:** Coronal T2-weighted MRI of the cervical and upper thoracic regions reveals multiple, well-defined hyperintense cystic lesions scattered extensively within the paravertebral and posterior cervical muscles. Numerous lesions demonstrate an eccentric mural nodule consistent with the scolex, confirming widespread myocysticercosis. The lesions are symmetrically distributed, with no associated vertebral body destruction or spinal cord compression. **Clinical Significance:** This image demonstrates the disseminated muscular form of cysticercosis, highlighting the extent of parasitic involvement in cervical and thoracic musculature. The absence of vertebral destruction or spinal cord compression underscores that the pathology is restricted to soft tissues, guiding therapeutic focus toward antiparasitic and supportive management rather than surgical intervention. Recognition of widespread muscular distribution is crucial for systemic evaluation and long-term monitoring. **Origin:** @GSVM Medical College, PMSSY Superspeciality Hospital, Kanpur, Uttar Pradesh, India. Pincode: 208002.

**Figure 5:**
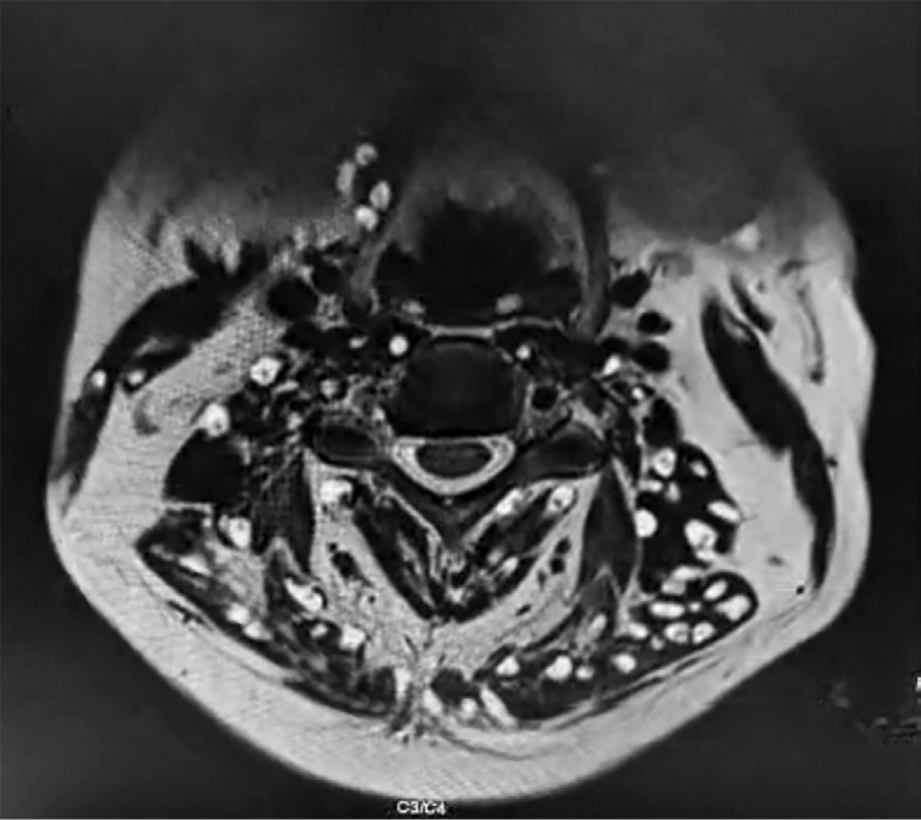
**Description:** Axial T2-weighted MRI at the C3–C4 vertebral level demonstrates multiple, well-defined hyperintense cystic lesions distributed within the paravertebral and posterior cervical musculature, many of these being with eccentric mural nodules representing the scolex. The cervical spinal cord and intervertebral discs are preserved without intramedullary signal changes. Findings are consistent with myocysticercosis involving cervical muscles. **Clinical Significance:** This axial view provides a cross-sectional perspective confirming muscular myocysticercosis with characteristic scolex identification. The preservation of spinal cord integrity and intervertebral disc morphology rules out compressive pathology. Differentiation from abscesses, neoplastic lesions, or inflammatory cysts is possible due to the presence of the scolex, which remains pathognomonic for cysticercosis. **Origin:** @GSVM Medical College, PMSSY Superspeciality Hospital, Kanpur, Uttar Pradesh, India. Pincode: 208002.

**Figure 6:**
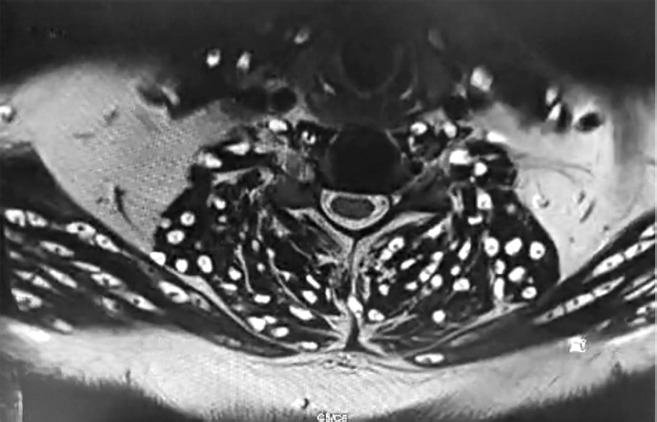
**Description:** Axial T2-weighted MRI at the C5–C6 level demonstrates numerous hyperintense cystic lesions embedded within the paravertebral and posterior cervical musculature. Several lesions reveal an eccentric mural nodule corresponding to the scolex, characteristic of myocysticercosis. The spinal cord appears preserved in contour and signal intensity, with no evidence of intramedullary pathology or compressive myelopathy. **Clinical Significance:** This axial section confirms advanced muscular involvement by cysticercosis at the mid-cervical level. The demonstration of scolex within multiple cysts provides a definitive diagnosis, separating it from other cystic or inflammatory muscular lesions. Preservation of spinal cord integrity and absence of myelopathy are reassuring findings that emphasize systemic medical management as the primary treatment approach. **Origin:** @GSVM Medical College, PMSSY Superspeciality Hospital, Kanpur, Uttar Pradesh, India. Pincode: 208002.

**Figure 7:**
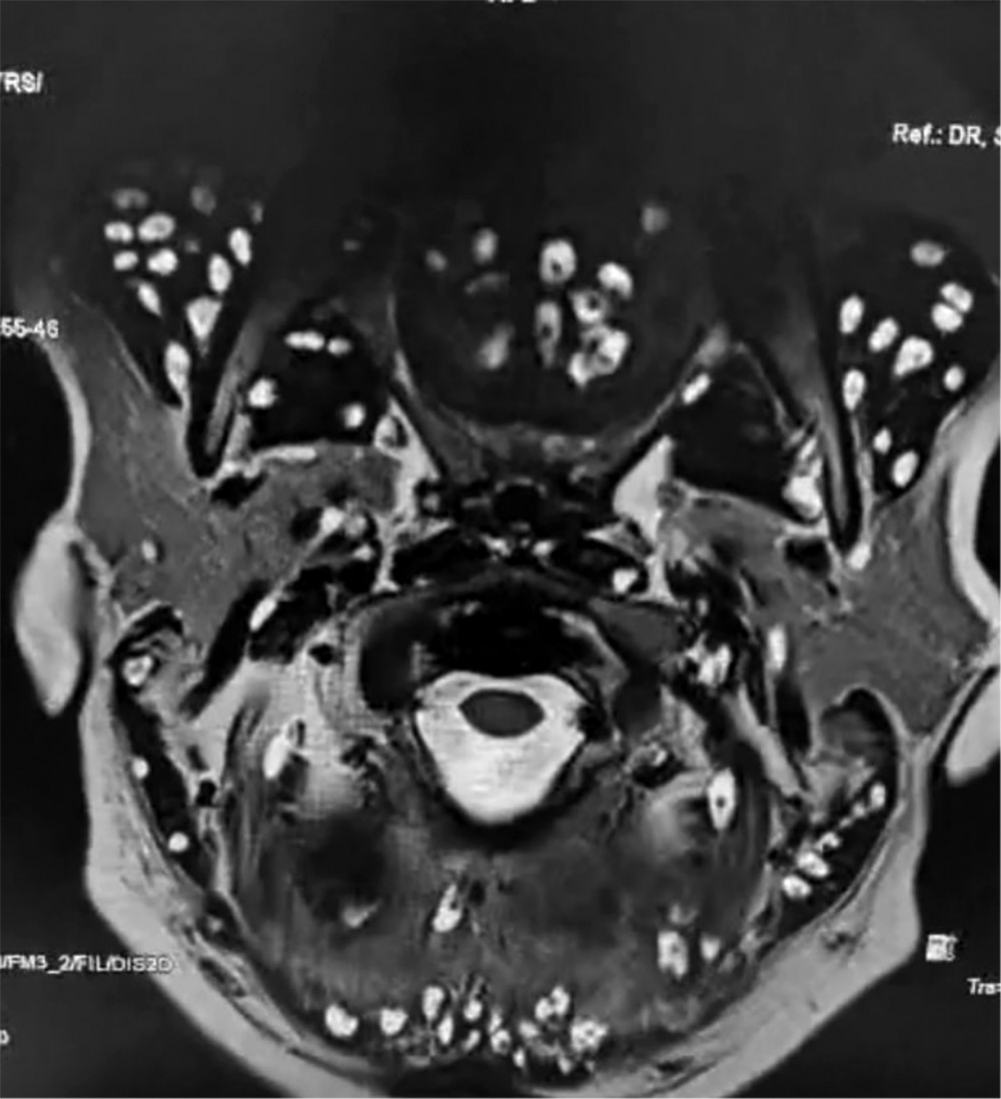
**Description:** Axial T2-weighted MRI at the upper cervical level demonstrates multiple, rounded hyperintense cystic lesions scattered within the paraspinal and posterior cervical musculature. Several lesions display an eccentric mural nodule, typical of the scolex, confirming myocysticercosis. The cervical spinal cord is centrally located with preserved signal intensity, and no evidence of compressive myelopathy or intramedullary involvement is identified. **Clinical Significance:** This axial view highlights early upper cervical involvement in disseminated myocysticercosis. The presence of a scolex within cystic lesions remains the hallmark diagnostic feature, distinguishing cysticercosis from other soft-tissue cystic masses. The absence of spinal cord compression and intramedullary changes indicates that the disease remains confined to musculature, allowing safe initiation of antiparasitic therapy with monitoring. **Origin:** @GSVM Medical College, PMSSY Superspeciality Hospital, Kanpur, Uttar Pradesh, India. Pincode: 208002.

Spinal MRI revealed reversal of cervical lordosis with diffuse disc bulges at levels C3/4, C4/5, C5/6, and C6/7, causing mild anterior thecal-sac indentation but without evidence of cord compression or myelomalacia (Figures 4–7) [5]. The lumbar spine showed disc desiccation with a diffuse bulge at L4–L5, indenting the thecal sac yet preserving normal vertebral body height and marrow signal intensity. No paravertebral abscess, vertebral destruction, or compressive myelopathy was noted [6].

Based on these findings, a final diagnosis of disseminated neurocysticercosis with muscular involvement and coexisting cervical and lumbar degenerative disc disease was made. The patient was treated with albendazole (15 mg/kg/day) and oral corticosteroids to reduce perilesional edema, along with antiepileptic therapy for seizure control. For spinal symptoms, conservative management with analgesics and physiotherapy was advised [7].

## Teaching Points

Neurocysticercosis as a common cause of seizures – in endemic regions, NCC should be suspected in patients presenting with new-onset seizures and chronic headache. Recognition of the scolex within cystic lesions on MRI is pathognomonic and crucial for diagnosis. Muscular cysticercosis, though uncommon, may present in extraocular, facial, and tongue muscles. Identifying the scolex differentiates it from abscesses or neoplastic lesions, preventing unnecessary invasive procedures. MRI remains the gold standard for detecting CNS and muscular involvement, delineating active disease, and excluding differential diagnoses such as tuberculomas or metastases [8]. Degenerative disc bulges, though incidental, may contribute to chronic pain or radiculopathy. Their recognition prevents misattribution of neurological symptoms solely to NCC. Optimal care requires antiparasitic therapy, seizure control, corticosteroids, and orthopedic/rehabilitative support for spinal degeneration.

## Final Diagnosis

Neurocysticercosis with Myocysticercosis was diagnosed.

## Limitations

This case report represents a single-patient observation; therefore, the findings cannot be generalized to a larger population. The diagnosis was based on characteristic MRI features without histopathological confirmation. Additionally, long-term follow-up imaging was not available to evaluate treatment response. Despite these constraints, the case provides valuable clinical and radiological insights into the coexistence of disseminated neurocysticercosis and degenerative spinal disease.

**Q1. What are the characteristic MRI findings that confirm neurocysticercosis?**

*Explanation:* On MRI, neurocysticercosis is identified by multiple ring-shaped lesions, each showing an eccentric nodule representing the scolex. These lesions are often accompanied by perilesional edema. In this case, their presence in the cerebral and cerebellar hemispheres provided a clear and definitive diagnosis.

**Q2. How is muscular (myocysticercosis) involvement identified in this patient?**

*Explanation:* Muscular involvement was detected through MRI, which revealed multiple cystic lesions within the extraocular, facial, and tongue muscles. The presence of an eccentric scolex inside these lesions confirmed that they were parasitic in origin, indicating disseminated myocysticercosis, which is an uncommon but clinically relevant finding.

**Q3. What was the significance of the cervical and lumbar disc pathology?**

*Explanation:* Imaging showed diffuse disc bulges at multiple cervical levels (C3–C7) and at the lumbar level (L4–L5). These changes caused indentation of the anterior thecal sac, consistent with early degenerative disc disease. Although unrelated to the parasitic infection, these findings explained the patient’s chronic pain and required attention to avoid future complications.

**Q4. What was the multidisciplinary management approach for this patient?**

*Explanation:* The patient was started on albendazole to target the parasitic infection, with corticosteroids prescribed to reduce associated inflammation and edema. Antiepileptic medication was given to control seizures. For spinal disc disease, a conservative plan including analgesics and physiotherapy was advised. This combined approach emphasized the importance of collaboration between neurology, infectious disease, and orthopedics in achieving optimal patient outcomes.
